# Anterior temporal face patches: a meta-analysis and empirical study

**DOI:** 10.3389/fnhum.2013.00017

**Published:** 2013-02-01

**Authors:** Rebecca J. Von Der Heide, Laura M. Skipper, Ingrid R. Olson

**Affiliations:** Department of Psychology, Temple UniversityPhiladelphia, PA, USA

**Keywords:** social networks, anterior temporal lobe, temporal pole, fMRI, social cognition, face processing, person memory, semantic memory

## Abstract

Evidence suggests the anterior temporal lobe (ATL) plays an important role in person identification and memory. In humans, neuroimaging studies of person memory report consistent activations in the ATL to famous and personally familiar faces and studies of patients report resection or damage of the ATL causes an associative prosopagnosia in which face perception is intact but face memory is compromised. In addition, high-resolution fMRI studies of non-human primates and electrophysiological studies of humans also suggest regions of the ventral ATL are sensitive to novel faces. The current study extends previous findings by investigating whether similar subregions in the dorsal, ventral, lateral, or polar aspects of the ATL are sensitive to personally familiar, famous, and novel faces. We present the results of two studies of person memory: a meta-analysis of existing fMRI studies and an empirical fMRI study using optimized imaging parameters. Both studies showed left-lateralized ATL activations to familiar individuals while novel faces activated the right ATL. Activations to famous faces were quite ventral, similar to what has been reported in previous high-resolution fMRI studies of non-human primates. These findings suggest that face memory-sensitive patches in the human ATL are in the ventral/polar ATL.

## Introduction

Although personally known and famous faces are often used interchangeably as “familiar” faces, there are distinct differences between them, which have implications on a theoretical and neural level. Personally familiar faces are associated with richer and more extensive first-person semantic and episodic knowledge than famous faces, as well as greater and more nuanced emotional significance (Sugiura et al., 2011), whereas famous faces are defined by their unique semantic attributes such as having starred in a popular movie or having served as president of the United States (Ross and Olson, 2011). fMRI studies of person memory have most commonly contrasted famous faces to unfamiliar faces and one of the most consistently reported activations is in the anterior temporal lobe (ATL); see Leveroni et al., 2000; Henson et al., 2003; Gobbini et al., 2004; Eger et al., 2005; Elfgren et al., 2006; Tsukiura et al., 2008; Trinkler et al., 2009; Brambati et al., 2010; Nielson et al., 2010; Ramon et al., 2010; Barense et al., 2011; Cloutier et al., 2011; Gesierich et al., 2011; Ross and Olson, 2011; Sugiura et al., 2011. These fMRI findings extend earlier PET studies reporting ATL activations to famous faces (Sergent et al., 1992a,b; Gorno-Tempini et al., 1998, 2000; Dubois et al., 1999; Gorno-Tempini and Price, 2001; Grabowski et al., 2001; Sugiura et al., 2001; Tsukiura et al., 2001; Damasio et al., 2004).

Rich and converging evidence for the role of the ATL in processing familiar faces also comes from the neuropsychology literature. Humans with ATL damage consistently exhibit problems with person memory, a disorder that Damasio and colleagues termed “amnesic associative prosopagnosia” (Damasio et al., 1990). Patients with focal unilateral lesions of the ATL due to stroke, insult, or resection surgery have difficulties remembering information about people, especially their names [reviewed by Olson et al. (2007); Simmons and Martin (2009); Wong and Gallate (2012)]. Damasio et al. (1990) carried out extensive research on the face processing capabilities of these individuals and reported that while face perception was intact, face memory was impaired. Left-lateralized lesions of the ATL tended to affect lexical aspects of person memory, such as recollection of names, while right-lateralized lesions tended to affect feelings of familiarity and processing and retrieval of biographical information.

Similarly, one of the first symptoms of semantic dementia, which is correlated with early deterioration of the ATLs, is difficulty remembering information about people such as their name and biographical information (Evans et al., 1995; Snowden et al., 2004). For instance, Thompson et al. (2003) reported that 31 of the 47 frontotemporal dementia patients in their study had specific complaints of difficulty recalling people's names.

The face sensitivity of the ATL is both mnemonic and perceptual. Electrophysiological and fMRI studies in humans and monkeys indicate that the ventral ATL is sensitive to novel faces (Nestor et al., 2011) and that activity in this region is enhanced by affective experience and conceptual familiarity with the depicted faces (Eifuku et al., 2010, 2011). As noted earlier, patients with ATL lesions have intact face perception, but impaired face memory (Damasio et al., 1990). Allison and colleagues recorded from the ventral surface of the human temporal lobe in patients undergoing resection surgery and found that a late event-related potential (ERP) termed the P350 localized to the ventral ATL was preferentially sensitive to faces and especially to face priming (Allison et al., 1999; Barbeau et al., 2007). Neurons in monkey ventral ATL have response profiles indicative of mnemonic activity: spike rate decreases rapidly with stimulus repetition, firing patterns are maintaining over brief delay intervals, and neurons appear to be sensitive to associations between faces and other stimuli (Nakamura et al., 1994; Nakamura and Kubota, 1996).

In sum, these findings provide consistent evidence that subregions of the ATL play an important role in person identification and memory. The goal of the current study was to examine this general finding in greater detail so that we can more precisely describe the functional anatomy and response properties of the ATL. We were interested in two specific questions: (1) Are the same ATL subregions sensitive to personally familiar and famous faces? (2) Are the face-sensitive subregions of the ATL localized to dorsal, ventral, lateral, or polar aspects of the ATL?

With respect to the first question, a small number of studies have reported greater BOLD activity to personally familiar as compared to famous faces in the ATL, as well as in the (MPFC), limbic regions, temporal parietal junction, and the posterior cingulate. These differences have been variously attributed to person-selective representations (Gobbini et al., 2004; Sugiura et al., 2006, 2008; Trinkler et al., 2009) or to the socioemotional aspects of recognizing familiar people (Gobbini et al., 2004; Gobbini and Haxby, 2007; Trinkler et al., 2009). In one recent study, repetition suppression to familiar faces was observed in a superior-medial region of the ATL (Sugiura et al., 2011). However, no activations to famous faces were found in the ATL. This finding is an outlier in the greater ATL literature since a large number of prior findings have reported that famous faces activate the ATL and that ATL damage impairs the ability to recognize or name famous faces. As such, more research is needed, using imaging parameters optimized for ATL coverage.

In regards to the second question it is informative to review findings from macaques in order to better understand the particular ATL subregions sensitive to faces. Several high-resolution fMRI studies in macaques have reported the existence of face-sensitive patches in the ventral ATL (Hadj-Bouziane et al., 2008; Moeller et al., 2008; Tsao et al., 2008; Bell et al., 2009; Rajimehr et al., 2009; Ku et al., 2011; Pinsk et al., 2011). Earlier single-unit studies reported face sensitive neurons in the anterior STS and the temporal pole (Hasselmo et al., 1989; Eifuku et al., 2004; De Souza et al., 2005; Leopold et al., 2006).

The monkey ATL face patches are found on the inferior bank of the anterior STS going into the anterior middle temporal gyrus (MTG) and the inferior surface of the ATL (Ku et al., 2011). No activations have been reported in the polar tip or superior aspects of the ATL. Two studies using unfamiliar face stimuli directly compared monkeys to humans and found homologous activated face patches in the anterior MTG and ventral surface of the ATL (Rajimehr et al., 2009; Pinsk et al., 2011). The majority of studies reporting ATL activations in humans have used famous or personally familiar face stimuli, although in two notable cases unfamiliar faces were shown to activate the ATL. Kriegeskorte et al. (2007) and Nestor et al. (2011) used multivariate techniques to ask which parts of the brain discriminate individual faces. The peak described by Kriegeskorte was in the right ventral ATL and the peak described by Nestor was in the right ventromedial ATL/uncus.

In contrast, the reported ATL activations to famous and personally familiar faces in human studies are typically in the polar tip and the superior ATL. It is unclear whether this localization is real or an artifact of methodological difficulties of imaging the ATLs. fMRI signals in the ATLs are compromised by susceptibility artifacts and signal distortion due to the proximity of these regions to the nasal sinuses and ear canals (Devlin et al., 2000). Moreover, many studies use a restricted field-of-view (FOV) that excludes the inferior parts of the brain, including the inferior ATL, from image acquisition (Visser et al., 2010).

In order to answer our two questions of interest, we used two methods. First, we conducted a meta-analysis of existing fMRI studies of person memory using the activation likelihood method (ALE) (Turkeltaub et al., 2002). Second, we conducted an fMRI study of person knowledge using imaging parameters optimized for ATL coverage in which activations to personally familiar individuals and famous faces were qualitatively compared to each other. We predicted that personally familiar as compared to famous and unfamiliar faces would be associated with greater activations in the superior-polar ATL due to the greater emotional processing performed on these stimuli. Evidence suggests the superior-polar aspects of the ATL are more closely connected with neuromodulatory regions such as the amygdala and hypothalamus, and are more greatly involved in abstract forms of social and emotional processing (see Olson et al., 2012 for a review). In addition we predicted that when optimized imaging parameters were used, we would find evidence of face-sensitive ATL activations that extend into the ventral ATL.

## Methods

### Meta-analysis

A total of 25 articles were included in the ALE analysis. Seven of these studies were used in the personally familiar condition with a total of 136 subjects (70 male, 66 female; mean age = 28.9 years). The famous face condition was comprised of 18 studies and had 247 subjects (125 male, 122 female; mean age = 28.74 years). A total of 202 foci were used in the personally familiar face condition and 340 foci were used in the famous face condition.

#### Procedure

Our methods follow those detailed by Binder et al. (2009) and are summarized below. All coordinates were reported in or converted to Talairach space. Random effects analysis [consistent with Eickhoff et al. (2009)] were conducted and probabilistic maps of the resulting sets of coordinates were constructed using the Activation Likelihood Estimate (ALE) method (Turkeltaub et al., 2002), implemented in the GingerALE 2.1 software package (Laird et al., 2005) (available at www.brainmap.org), using an 8-mm FWHM 3D Gaussian point spread function and a spatial grid composed of 2 × 2 × 2 mm voxels. This method treats each reported focus as the center of a Gaussian probability distribution. The 3D Gaussian distributions corresponding to all foci included in a given random effects analysis are summed to create a whole-brain map that represents the overlap of activation peaks at each voxel. The ALE statistical map is converted into a voxelwise probability map. ALE maps from each dataset were thresholded at an ALE value that yielded a corrected mapwise value and a false discovery rate (FDR) of *p* < 0.05. The maps depicted in the figures are the corrected ALE maps generated by GingerALE 2.1 software.

#### Study inclusion criteria and description

Studies were identified through searches of online databases for the years 1980 through 2012 (see Table 1). Any additional relevant articles known to the authors, cited in the initial set of articles, or encountered during the review process were added to the list. Inclusion criteria were: (1) the use of fMRI; (2) testing of healthy young human participants; (3) use of a standard control or baseline task; (4) whole or nearly whole brain analysis; (5) availability of peak activation coordinates from a group activation map; and (6) use of several different famous or personally familiar faces as stimuli (studies that used a single face stimulus, such as a romantic partner, were excluded). Studies were also excluded if they used non-standard cohort sizes (*N* < 10) or imaging parameters (e.g., TE of 66) that diminished the possibility of observing activations in the ATLs. Our analysis used two person memory comparisons:

1. Personally known faces (personal acquaintances, close friends, and familiar faces): Total papers = 7. Coordinates were included from the following contrasts: friends/family > baseline; friends/family > unfamiliar.
2. Famous faces: Total Papers = 18. Coordinates were included from the following contrasts: famous > baseline; famous > unfamiliar.

**Table 1:** **fMRI studies of person knowledge included in the random effects ALE analysis**.

**References**	***N* (Males)**	**Mean age**	**Face stimuli**			**ATL activations?**	**Task**
			**Personally familiar**	**Trained knowledge**	**Famous**		
Arsalidou et al., 2010	10 (4)	35.4	x			No	Familiar vs. Celebrity vs. Stranger faces
Bai et al., 2011	21 (11)	33			x	Bilateral ATL	Famous vs. Unfamiliar faces
Barense et al., 2011	18 (6)	27.3			x	Bilateral ATL	Odd-one-out task: Familiar vs. Unfamiliar faces
Bernard et al., 2004	12 (3)	58.7			x	No	Famous vs. Non-famous faces
Brambati et al., 2010	12 (4)	23			x	Right ATL	Semantic judgment task: Famous vs. Scrambled famous faces
Cloutier et al., 2011	19 (0)	18.9		x		Right ATL	Trained familiar faces task
Donix et al., 2010	12 (6)	30.4	x			No	Familiar vs. Unfamiliar faces
Eger et al., 2005	15 (4)	21.8			x	Bilateral ATL	Repetition suppression task: Familiar vs. Unfamiliar faces
Elfgren et al., 2006	15 (7)	23.3			x	Bilateral ATL	Famous vs. Unfamiliar faces
Gesierich et al., 2011	21 (7)	28.4			x	Bilateral ATL	Famous vs. Scrambled faces
						Left ATL	Famous vs. Non-famous Faces
Gobbini et al., 2004	10 (5)	26.8	x		x	Right ATL	Familiar vs. Famous vs. Unfamiliar faces
Henson et al., 2003	18 (10)	28 (median)			x	Left ATL	Famous vs. Non-famous faces
Ishai et al., 2002	9 (5)	27			x	No	Famous vs. scrambled faces
Leveroni et al., 2000	11 (5)	32			x	Right ATL (Famous)	Newly-learned vs. Famous vs. Unfamiliar faces
Nielson et al., 2010	17 (10)	28.8			x	Left ATL	Famous vs. Non-famous faces
Pourtois et al., 2005	13 (8)	26			x	RightATL	Repetition suppression: Famous vs. Non-famous faces
Ramon et al., 2010	13 (5)	23	x			Right ATL	Semi-familiar vs. Computer generated unfamiliar faces
Ross and Olson, 2011	11 (4)	23		x	x	Bilateral ATL (famous); Left ATL (trained)	Non-famous faces and places paired with semantic information
Rothstein et al., 2005	14 (7)	28			x	Bilateral ATL	Famous vs. Morphed famous faces
Sugiura et al., 2005	28 (16)	19–31	x			Left ATL (personally known)	Own vs. Familiar vs. Unfamiliar faces
Sugiura et al., 2011	34 (26)	18–26	x		x	Bilateral ATL (personally known)	Familiar vs. Unfamiliar vs. Famous faces
Trinkler et al., 2009	14 (8)	20–23	x		x	Bilateral ATL (personally known); Right ATL (famous)	Famous vs. Familiar vs. Unfamiliar faces
Tsukiura et al., 2006	11 (7)	21.5		x		Right ATL	Trained familiar with semantic information vs. Trained familiar without semantic information
Tsukiura et al., 2003	11 (11)	22.3		x		Bilateral ATL	Trained familiar faces vs. Mosaic faces vs. Fixation
Tsukiura et al., 2008	10 (6)	22.1		x		Right ATL	Trained familiar vs. Unfamiliar faces
Turk et al., 2005	13 (6)	24			x	No	Semantic task during famous face viewing
*N*			7	5	18	21/26	

Data from the “trained knowledge” column includes studies in which study participants were trained to associate different types of semantic knowledge (e.g., a profession) with a novel face. These data were not included in an ALE analysis because the number of studies was small and the training paradigms variable. The last two columns list whether ATL activations were reported and the experimental task/comparisons in each study. We note that Turk and colleagues (2005) reported that technical limitations kept them from seeing activations in the ATL.

We created conjunction maps by overlaying these analyses.

### Empirical study

#### Participants

Seventeen female adults were recruited from the greater Philadelphia area via local advertisements. Data from two participants were excluded due to excessive movement. The final sample consisted of 15 female participants (mean age = 22.33, SD = 3.51). We chose to restrict our sample to females because numerous behavioral and neural studies of face recognition have reported significant gender differences (Ellis et al., 1973; Killgore and Yurgelun-Todd, 2001; Lewin and Herlitz, 2002; Rehnan and Herlitz, 2006, 2007; McBain et al., 2009; Ino et al., 2010; Megreya et al., 2011). All participants received monetary compensation for their participation. They were native English speakers, right-handed, had normal hearing and normal or corrected-to-normal vision, and no history of psychological, developmental, or neurological disorders. Informed consent was obtained according to the guidelines of the Institutional Review Board of the Temple University.

#### Stimuli

There were four types of stimuli. The *famous face stimuli* (=Faces_famous_) consisted of 32 people that had a high likelihood of being known by the average American undergraduate or graduate student (e.g., Brad Pitt). The *friend face stimuli* (=Faces_friends_) were individually tailored for each participant. Each participant brought two different photos of the faces of 5 best friends, 5 close friends, and 5 acquaintances to a pre-testing session. Photos consisted of snapshots that varied in lighting, poses, facial expressions, quality, etc. The *unfamiliar face stimuli* (=Faces_novel_) were a wide selection of unknown faces whose ethnicities and ages matched that of the famous faces and the friends. Like the other face stimuli, they consisted of a mixture of professional photographs and snapshots that varied in lighting, pose, etc. Last, the *baseline control stimuli* consisted of blurred images. Additional comparison and baseline conditions consisted of famous landmarks (=Landm_famous_), non-famous landmarks (=Landm_novel_), and a blurred landmark baseline. Similar stimuli were used as a fame localizer in a previous study by our laboratory (Ross and Olson, 2011).

#### fMRI task and design

All participants were provided with standardized computer-based instructions and a practice session prior to the scan. During the scan, a ~10 min long high-resolution anatomical scan was collected prior to the experiment. Portions on the fMRI task are irrelevant to the question asked in this study and will be described in a different publication.

***Famous face task.*** Our laboratory used a face memory localizer task that had been used successfully in a previous study to localize activation for famous faces (Ross and Olson, 2011). During the localizer run, a 0-back task was used in which participants were told to press a button with their left index finger when two stimuli of the same type were detected in succession: both famous, both non-famous, or both baseline images in succession. Participants were also told to press with the right index finger when it was a mixed condition (e.g., one famous and one non-famous face). During one block two stimuli were presented in succession, for the duration of 4.5 s each. Each blocked presentation was preceded by a 3 s prompt with a brief reminder of the instruction and followed by a 3 s response prompt. Blocks consisted of pairs of pictures that were both famous (20), both non-famous (20), mixed (10), face baseline (5), landmark baseline (5) totaling 60 blocks, and a duration of 15 min.

***Friend face task.*** There were five experimental runs during which participants performed a social and geographical closeness task using photos of friends and unknown individuals. The experimental task had a 2 (type of closeness: social, geographical) × 2 (stimulus: faces, names) × 3 (distance: close, medium, far) design. Baseline conditions were also included in each run and consisted of the faces and names of unknown people. Only brain activations in response to the faces of friends and unknown people in the social closeness and baseline conditions are relevant to the question of interest in this study and are described in greater detail. Photographs of the faces of “best friends,” “close friends,” “acquaintances,” and “unknown people” were presented in separate blocks during a single run.

On each trial one photo was presented left of center and the second photo was presented right of center. One photo in each pair was always presented at a discriminable location above the center x axis of the screen and the other photo was presented at a counter location below the x axis. During the social closeness condition, participants indicated by button press whether the friend shown on the left or on the right of the screen was socially closest to them. During baseline blocks, participants were asked to decide which photograph of an unknown person was presented in a higher position on the screen by pressing the left or right button with their index finger. Each run was comprised of 14 blocks that were 18 s long. A 3 s instruction screen was followed by 5 trials consisting of a 2.5 s presentation of two friends photographs and a 0.5 s inter-trial interval. A 9 s rest period during which a fixation cross was presented followed each block. Each condition was presented one time in a single run.

#### Imaging procedure

Neuroimaging sessions were conducted at the Temple University Hospital on a 3.0 T Siemens Verio scanner (Erlangen, Germany) using a 12-channel Siemens head coil.

**Figure 1:**
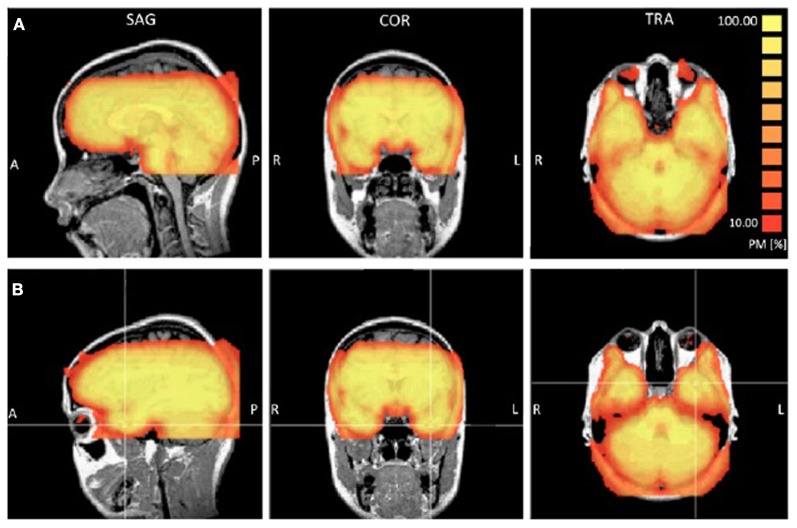
**Probability maps (PM) reporting the percentage or participants (ranging from 10 to 100%) that showed a TSNR greater than 40 for each voxel.** Panel **(A)** provides a view of the coverage for all participants in the orbitofrontal cortex. Panel **(B)** provides a view of the coverage in the left anterior temporal lobe.

Functional T2^*^-weighted images sensitive to blood oxygenation level-dependent contrasts were acquired using a gradient-echo echo-planar pulse sequence [repetition time (TR), 3 s; echo time (TE), 20 ms; *FOV* = 240 × 240; voxel size, 3 × 3 × 2.5 mm; matrix size, 80 × 80; flip angle = 90°] and automatic shimming. This pulse sequence was optimized for ATL coverage and sensitivity based on pilot scans performed for this purpose, details of which are reported in Ross and Olson (2010). Visual inspection of the coregistered functional image confirmed excellent signal coverage in the ATLs in all participants. Some signal loss in the orbitofrontal cortex was observed and varied between participants (see Figure 1).

Thirty-eight interleaved axial slices with 2.5 mm thickness were acquired to cover the temporal lobes. On the basis of the anatomical information of the structural scan the lowest slice was individually fitted to cover the most inferior aspect of the inferior temporal lobes.

The functional runs were preceded by a high-resolution anatomical scan that was ~10 min long. The anatomical image was used to fit the volume of covered brain tissue acquired in the functional scan. The T1-weighted images were acquired using a three-dimensional magnetization-prepared rapid acquisition gradient echo pulse sequence (TR, 1900 ms; TE, 2.94 ms; FOV = 188 × 250 mm; inversion time, 900 ms; voxel size, 1 × 0.9766 × 0.9766 mm; matrix size, 188 × 256; flip angle = 9°, 144 contiguous slices of 0.9766 mm thickness). Visual stimuli were shown through goggles with a resolution of 800 × 600 pixels, purchased from Resonance Technologies, CA, USA. Responses were recorded using a four-button fiber optic response pad system. The stimulus delivery was controlled by E-Prime software (Psychology Software Tools Inc., Pittsburg, PA) on a windows laptop located in the scanner control room.

#### Image analysis

fMRI data were preprocessed and analyzed using Brain Voyager software (Goebel et al., 1998). The preprocessing of the functional data included a correction for head motion (trilinear/sinc interpolation), the removal of linear trends and frequency temporal filtering using a fast fourier transform (FFT) and a cut-off of three cycles or sine waves. The data were coregistered with their respective anatomical data and transformed into Talairach space (Talairach and Tournoux, 1988). The resulting volumetric time course data were smoothed using an 8 mm Gaussian kernel.

For all blocks, a canonical hemodynamic response function (HRF) was modeled spanning the 15 s for each block. A z-transform was applied to normalize the time course. Predictors were built by convolving the boxcar waveform for each condition with a double-gamma HRF (onset = 0, response undershoot ratio = 6, time to response peak = 5 s, time to undershoot peak = 15 s, response dispersion = 1, undershoot dispersion = 1). Only the manipulated conditions in the 2 × 3 × 3 design were included in the model. Motion parameters were not included as covariates in the regression, because motion was corrected for in preprocessing. Including them as covariates in the regression has been shown to have a deleterious effect on the mean contrast estimates in block design studies (Johnstone et al., 2006). The 3 s instruction screen at the start of each block and the 9 s rest period following each block were modeled out and were also not included in the HRF.

#### Temporal signal to noise ratio (TSNR)

The ATL and inferior portions of the frontal lobe are prone to susceptibility artifacts so we examined [Temporal Signal to Noise Ratio (TSNR)] maps to ensure that the quality of the signal in these regions was adequate to detect BOLD signal. To check the consistency of coverage in the signal quality in the ATLs, we generated probability maps indicating for each voxel the percentage of participants that showed TSNR above the threshold of 40, which is considered sufficient for detecting differences between conditions (Murphy et al., 2007). Using a highly conservative estimate of signal coverage in the bilateral ATL, 7 participants showed full coverage and 8 participants showed some signal loss either on the inferior surface (a small portion of the inferior ATL), medial surface near piriform cortex, or a region just posterior to the ATLs caused by the ear canals. Six participants showed full coverage of the orbitofrontal cortex and 9 participants showed minor loss in coverage of the most inferior slice in the posterior orbitofrontal cortex. Overall, the signal quality in the ATL was excellent. Figure 1 shows a probability map that the TSNR was above 40 for each voxel for all participants.

#### Data analysis

We performed a whole-brain analysis using a random effects general linear model for conditions in each of the experimental tasks. We used a voxelwise FDR approach to try and replicate previous findings in humans for famous/non-famous faces and landmarks. We also used this approach to assess activations in the ATL for novel faces/novel landmarks that were comparable to those activations reported in monkey studies. Second, we conducted somewhat more liberal whole-brain analyses using a cluster thresholding procedure that calculates the likelihood of obtaining different cluster sizes over 1000 trials. This procedure began by setting the voxelwise threshold to *p* < 0.05 and was followed by the Monte Carlo simulation of this data to produce a cluster threshold that also ensures a global error rate of *p* < 0.05. Each cluster size threshold was calculated within a whole-brain gray matter map.

## Results

### ALE results

**Figure 2:**
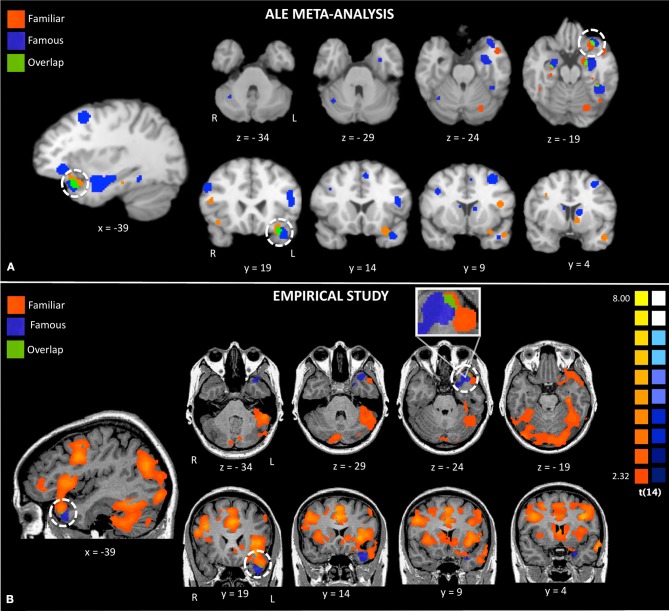
**Activations to famous and familiar faces from a random effects ALE meta-analysis (Panel A) and activations from random effects group analyses in the empirical study (Panel B).** The white circles highlight activations to famous and familiar faces in the left anterior temporal lobe.

The results of the ALE random effects analyses are reported in Table 2 and depicted in Figure 2, top panel. All ALE results were corrected for multiple comparisons using the FDR method at a threshold of *p* < 0.05 and a minimum cluster size of 100 mm^3^. Both conditions showed a left-lateralized bias although some significant activations were also found in the right hemisphere. Of interest to the topic of this paper, there were three left-lateralized ATL loci's: (1) a ventromedial activation, corresponding to entorhinal cortex to famous faces; (2) an anterior STS activation to personally familiar faces; and (3) a temporal pole [anterior superior temporal gyrus (STG)] activation to both famous and personally familiar faces. The entorhinal activation was part of a larger activation extending along the entire length of the hippocampus, spilling over into the amygdala. In contrast, the anterior STS activation was very focal. Last, the striking temporal polar activation continued into orbitofrontal cortex in more dorsal slices. Famous faces showed a relatively more ventral pattern of activation with the most ventral activation in the medial ATL/entorhinal cortex at −26, −6, −29.

**Table 2:** **Talairach coordinates of peak activations from the random effects ALE meta-analysis**.

**Comparison**	**Region**	***x***	***y***	***z***	**BA**
**FAMOUS FACES vs. BASELINE**					
Frontal regions	L Inferior frontal gyrus	−46	26	12	45/47
	L Medial frontal gyrus	−6	56	2	10
	L Orbitofrontal cortex	−34	18	−22	47
	R DLPFC	50	20	30	8/9
	L Anterior cingulate	−8	40	−8	24
Temporal regions	L Anterior lateral STG	−50	−10	−10	22
	R Anterior STG	54	−4	−8	22
	L Posterior MTG	−52	−36	−4	21
	L Amygdala	−20	−10	−12	–
	R Amygdala	26	−12	−12	–
	L Medial parahippocampal gyrus	−26	−20	−14	28
	L Fusiform gyrus; e.g., “FFA”	−40	−48	−14	37
	R Fusiform gyrus; e.g., “FFA”	34	−52	−10	37
Occipital regions	R Lingual gyrus	40	−74	−6	18
	R Cuneus	22	−84	18	17
	L Dorsal/posterior cingulate	0	−38	30	31/23
Other	R Cerebellum	36	−60	−26	–
	L Caudate body	−12	4	12	–
**FAMILIAR FACES vs. BASELINE**					
Frontal regions	L DLPFC	−4	46	26	8/9
	L Orbitofrontal cortex	−28	16	−18	47
	R Inferior frontal gyrus	46	26	12	45
	L Cingulate gyrus	−4	0	34	24
	L Insula	−42	10	16	–
	R Insula	48	−6	−2	13
Temporal regions	L Temporal pole/STG	−46	6	−22	38
	R Posterior STG	48	−54	12	39
	L Parahippocampal gyrus	−24	−16	−16	28
	R Parahippocampal gyrus	30	−28	−14	28
	L Amygdala	−18	−8	−16	–
	L Fusiform gyrus; e.g., “FFA”	−36	−42	−16	20
Parietal/occipital regions	L Posterior cingulate/precuneus	2	−52	12	29/31
Other	L Cerebellum	−22	−72	−22	–
	L Medial globus pallidus	−8	0	−2	–
**FAMOUS ∩ FAMILIAR FACES**					
Frontal regions	L Middle frontal gyrus	−30	8	48	6
	R Middle frontal gyrus	36	8	34	6/46
	L Medial frontal gyrus	−6	56	2	10
	L Superior frontal gyrus	−18	30	44	8
	L Orbitofrontal cortex	−32	18	−20	47
	L Inferior frontal gyrus/insula	−48	28	12	45
	L Cingulate gyrus	0	−38	30	–
	L Anterior cingulate	−6	36	12	24
Temporal regions	L Anterior STG	−52	−8	−10	22
	L Temporal pole/STG	−44	4	−24	38
	R Posterior STG	48	−54	12	39
	R Anterior lateral STG	52	−2	−8	22
	L Posterior MTG	−46	−62	20	39
	R Posterior MTG	48	−60	24	39
	L Amygdala	−20	−10	−12	–
	L Inferior medial TP/parahippocampal gyrus	−28	−8	−22	–
	L Fusiform gyrus; e.g., “FFA”	−40	−48	−14	37
	R Fusiform gyrus; e.g., “FFA”	34	−52	−10	37
Parietal/occipital regions	R Lingual gyrus	40	−74	−6	18
	L Posterior cingulate	−4	−56	12	29
Other	L Caudate	−20	30	4	–
	R Medial globus pallidus	24	−12	−12	–
	L Thalamus midline nucleus	−8	−16	14	–

### Empirical study results

#### Behavioral findings

During the famous face task, participants correctly identified 94% of the famous faces, 70% of the non-famous faces, 86% of the famous landmarks, and 67% of non-famous landmarks. Mixed face trials (distractors) were identified with 78% accuracy and mixed place trials in 71% of cases. The baseline task was correctly performed in 92% of all cases.

The social closeness judgments participants made of their best friends during the five main experimental runs were 91% consistent with their previously reported rank orderings. Response consistency was judged by comparing the behavioral data during the scanner task to the rank orderings of best friends on social closeness that participants made during the preliminary testing session. Participants were 98% accurate performing the baseline task with photos of unknown people.

#### Functional MRI results

First we performed a “sanity check” that was aimed at replicating prior findings about non-famous faces/landmarks. We asked whether faces and landmarks *per se* activated well-known networks of brain regions including the fusiform face area. We performed a whole brain analysis of the conditions in the famous face task using a random effects general linear model. We then conducted a contrast between all faces and landmarks [(Faces_famous_ + Faces_novel_ + Faces_mixed_) vs. (Landm_famous_ + Landm_novel_ + Landm_mixed_)] at an FDR corrected threshold [q_(FDR)_ < 0.05; *p* < 0.006] for the whole brain. The *t*-map revealed that faces relative to landmarks-activated regions previously shown to be engaged in face processing: right FFA, bilateral temporoparietal junction going into the posterior STS, precuneus, bilateral ATL in the anteriormost section of the MTG, bilateral entorhinal cortex, and a small region of MPFC. Likewise, landmarks engaged a typical pattern of activity in bilateral PPA, retrosplenial cortex, and regions of the dorsal visual stream. At this threshold, landmarks did not activate the ATL.

**Figure 3:**
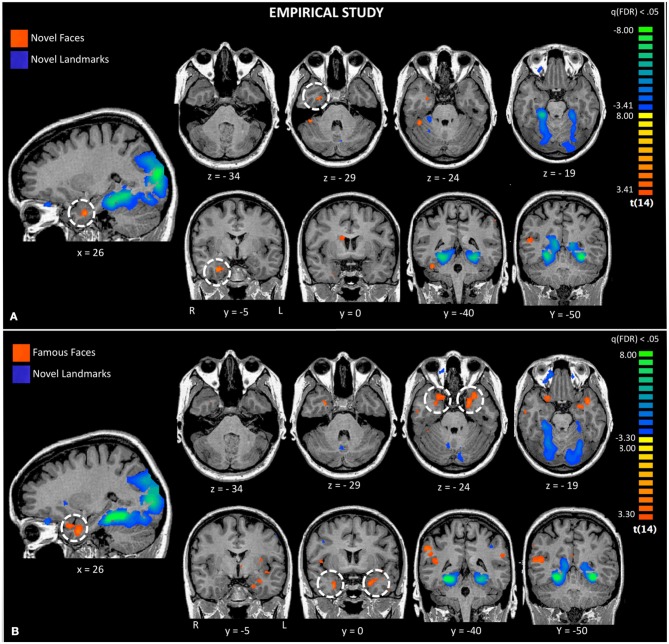
**Activations to a comparison of novel faces and novel landmarks from the empirical study are also shown (Panel A).** The white circle highlights activation to novel faces in the right ATL. Activations to a comparison of famous faces and novel landmarks from the empirical study are also shown (Panel **B**). The white circle highlights activation to famous faces in the left and right ATL.

Second, we asked whether novel faces, as used in many monkey fMRI studies, activate the ATL. We compared novel faces to novel landmarks [Faces_novel_ vs. Landmarks_novel_; q_(FDR)_ < 0.05; *p* < 0.005] and found a small region of activation in the right FFA and the right ventromedial ATL in a region corresponding to perirhinal cortex (see Figure 3, top panel). This ATL activation is posterior to the ATL activations to famous and familiar faces, reported later. Landmarks engaged a typical pattern of activity in bilateral PPA, retrosplenial cortex, regions of the dorsal visual stream, and did not activate the ATL.

Third, using the same baseline task as the prior analysis, we asked whether famous and personally familiar faces activated the ATL. We compared famous faces to novel landmarks [Faces_Famous_ vs. Landmarks_novel_; q_(FDR)_ < 0.05; *p* < 0.005] and found regions typically engaged in face processing including the right FFA, precuneus, MTG, and bilateral ventromedial ATL activations. Although the centroid of the ATL activity was similar to that found for novel faces, the activations were bilateral and more extensive (see Figure 3). Again, landmarks demonstrated a typical pattern of activity activating the bilateral PPA, retrosplenial cortex, regions of the dorsal visual stream, and did not activate the ATL.

Fourth, to specifically examine face familiarity, we asked whether famous faces preferentially activated the ATL using the contrast (Faces_famous_ vs. Faces_novel_). We present these results corrected for multiple comparisons at *p* < 0.05 with a cluster threshold applied (cluster threshold = 45). This contrast revealed two face patches in the left ATL: (1) a patch in the polar aspect of the MTG; (2) a smaller medial activation in entorhinal cortex. One right-lateralized ATL activation, in the ventral ATL corresponding to parahippocampal gyrus/entorhinal cortex, was found when we used a more liberal threshold of *p* < 0.05 (uncorrected). Other activations specific to famous faces were in the posterior cingulate, retrosplenial cortex, left FFA, and right supermarginal gyrus. Non-famous faces compared to famous faces preferentially activated clusters in the parietal and frontal lobes (see Table 3).

**Table 3:** **Talairach coordinates of peak activations from the empirical study**.

**Comparison**	**Regions**		***x***	***y***	***z***	**BA**
Faces > Landmarks	Frontal	L Inferior frontal gyrus	−18	19	−18	47
		R Orbitofrontal cortex	20	11	−20	47
		R Medial frontal gyrus	8	43	24	9
		L Medial frontal gyrus	−4	55	1	10
	Temporal	L Fusiform gyrus; e.g., “FFA”	−40	−50	−20	37
		R Fusiform gyrus; e.g., “FFA”	40	−41	−23	37
		L Temporal pole/STG	−41	9	−29	38
		R Temporal pole/STG	32	6	−26	38
		L MTG	−37	4	−31	21
		R MTG	45	4	−26	21
		L Parahippocampal gyrus	−23	2	−21	28
		R Parahippocampal gyrus	29	7	−21	28
		R Amygdala	21	−5	−11	–
		L STG	−53	−49	15	22
		R STG	51	−45	14	22
	Parietal	Precuneus	0	−60	28	31
Landmarks > Faces	Temporal	L Parahippocampal place area	−25	−37	−13	36
		R Parahippocampal place area	24	−32	−15	36
		L Retrosplenial cortex	−13	−54	7	30
		R Retrosplenial cortex	12	−55	8	30
	Occipital	L Medial occipital gyrus	−31	−85	19	19
		R Medial occipital gyrus	27	−84	19	19
		Cingulate gyrus	0	−44	41	31
Famous faces > Novel faces	Temporal	L Temporal pole/STG	−32	17	−29	38
		R Insula	56	−36	16	13
		Cingulate gyrus	−3	−15	39	24
		Posterior cingulate	0	−39	4	–
		R Retrosplenial cortex	15	−45	3	30
Novel faces > Famous faces	Frontal	L Medial frontal gyrus	−33	51	6	10
		R Medial frontal gyrus	27	50	8	10
		L Precental gyrus	−45	15	−39	38
		R Precentral gyrus	46	15	−39	38
Novel faces > Novel landmarks	Temporal	R Anterior parahippocampal gyrus	25	−4	−28	36
		R Fusiform gyrus; e.g., “FFA”	40	−39	−27	37
		R MTG	53	−21	−6	21
		Anterior cingulate	0	43	4	32
		R STG	49	−53	14	39
	Parietal	Precuneus	0	−53	24	31
Novel landmarks > Novel faces	Temporal	L Parahippocampal place area	−25	−38	−13	36
		R Parahippocampal place area	22	−32	−16	36
		L Retrosplenial cortex	−14	−50	7	30
		R Retrosplenial cortex	14	−55	10	30
	Parietal	Postcentral gyrus	61	−9	15	43
	Occipital	L Middle occipital gyrus	−31	−82	20	19
		R Middle occipital gyrus	29	−82	15	19
Famous faces > Novel landmarks	Frontal	R Orbitofrontal cortex	20	12	−17	47
		R Insula	42	1	6	13
		Anterior cingulate	−9	32	−3	32
		L Medial frontal gyrus	−3	57	6	10
		R Medial frontal gyrus	3	57	8	10
	Temporal	L ATL	−30	10	−24	38
		R ATL	25	6	−24	38
		R ITG	54	−10	−24	20
		R Fusiform gyrus; e.g., “FFA”	39	−42	−24	37
		L MTG	−49	0	−34	21
		R MTG	39	−13	−5	21
	Parietal	Precuneus	0	−60	26	31
		Posterior cingulate gyrus	0	−16	40	24
	Occipital	Medial occipital gyrus	0	−89	12	18
		Middle temporal gyrus	52	−66	8	37
Novel landmarks > Famous faces	Frontal	L Insula	−35	19	2	13
		R Insula	35	17	2	13
		L Precentral gyrus	−44	12	37	9
		R Precentral gyrus	40	9	33	9
	Temporal	R Parahippocampal place area	29	−36	−11	36
		R Parahippocampal place area	−28	−48	−11	36
		L Retrosplenial cortex	−15	−55	7	30
		R Retrosplenial cortex	15	−55	9	30
	Occipital	L Medial occipital gyrus	−27	−84	7	18
		R Medial occipital gyrus	30	−81	10	18
Famous landmarks > Novel landmarks	Occipital	Occipital gyrus	0	−89	16	18
Novel landmarks > Famous landmarks	Frontal	R Inferior frontral gyrus	29	13	−11	13
		L Medial frontal gyrus	−2	27	36	6
		R Inferior frontal gyrus	53	21	26	9
		R Precentral gyrus	37	7	35	9
		R Inferior frontal gyrus	37	16	4	45
		R SFG	29	55	16	10
	Temporal	R Parahippocampal place area	27	−44	−9	37
		L Parahippocampal place area	−29	−46	−9	37
	Occipital	R Inferior occipital gyrus	31	−88	−6	18
Familiar faces > Novel faces		Left temporal pole/STG	−46	12	−23	38
		Lateral inferior frontal gyrus	−33	25	−11	47
		Medial frontal gyrus	0	10	47	32
		Precuneus	0	−61	23	23
		L Fusiform face area	−38	−49	−16	37
		R Fusiform face area	34	−48	−18	37
Famous ∩ Familiar faces	Temporal	L Temporal pole/STG	−38	20	−25	38
		Posterior cingulate/precuneus	−3	−42	4	29/31

To see whether the ATL activations were specific to famous face stimuli as compared to other unique stimuli such as famous landmarks, we performed the same contrast corrected for multiple comparisons but on landmark stimuli (Landmarks_famous_ vs. Landmarks_novel_; *p* < 0.05 cluster threshold = 39). Bilateral ATL activations were observed in a ventral medial portion of the ATL, more posterior and ventral in the uncus/perirhinal cortex (−22, −1, −39) to that observed in the famous face contrast. A similar locus of activity was reported previously (Ross and Olson, 2011). Activations to famous landmarks did not overlap with the activations to famous faces in the ATLs.

Last, we asked whether personally familiar individuals (e.g., best friends) activated the ATL in a similar manner as famous faces. We performed a whole brain analysis of conditions in the friend face task using a random effects general linear model. We then conducted a contrast corrected for multiple comparisons: Faces_friends_ vs. Faces_novel_; *p* < 0.05; cluster threshold = 51. This contrast showed two left-lateralized ATL patches: (1) a patch in the anterior STS; and (2) a patch in the anterior MTG/pole. Additional left lateralized activations were observed in orbitofrontal cortex, medial PFC, ACC, hippocampus, precuneus, extrastriate visual cortex, as well as bilateral activations in the FFA, TPJ, and other regions (see Table 3). No regions were relatively more sensitive to novel faces.

The conjunction map revealed minor overlap for famous and familiar in the left ATL polar tip (see Figure 2, bottom panel).

Visual comparison between the activations found in the meta-analysis and our empirical study revealed strikingly similar activations (see Figure 2). Both the ALE and empirical analyses showed left-lateralized activations to famous and personally familiar faces in the ATL. Within the left ATL, both analyses showed that famous faces active a ventromedial region corresponding to entorhinal cortex. Also both analyses showed that famous and personally familiar faces activate an overlapping region of the temporal pole (BA 38). In the ALE analysis, this extended into orbitofrontal cortex for both comparisons while in the empirical analysis it was only observed for the personally familiar contrast. One difference revealed by this comparison is that activations to famous faces were relatively more ventral in the empirical analysis (z plane = −34) as compared to the ALE analysis (z plane = −29).

## Discussion

The goal of the present study was to investigate two questions in order to learn more about the sensitivity of the ATL to faces: (1) Are the same ATL subregions sensitive to personally familiar, famous, and novel faces? (2) Are the face-sensitive subregions of the ATL localized to dorsal, ventral, lateral, or polar aspects of the ATL? To answer these questions we qualitatively compared the results of our ALE meta-analysis of face familiarity to the findings from our empirical fMRI study using famous, personally familiar, and unfamiliar faces. We were particularly interested in whether ATL activations to different categories of faces were present in the ventral ATL, similar to what has been reported in monkeys using novel and familiar faces. In order to maximize our ability to find activations in the ATL in our empirical study, we used imaging parameters optimized for ATL coverage. Overall, the findings of the empirical study and meta-analysis were notably consistent revealing left-lateralized ATL sensitivity to two kinds of familiar faces and right-lateralized sensitivity to novel faces. In addition, the activations to familiar faces associated with knowledge were found in distinct subregions of the left ATL with the only overlap occurring in the polar tip.

### Activation of the anterior temporal lobes by novel, famous, and personally familiar faces

We began by conducting a meta-analysis of existing fMRI studies of person memory where the typical subtraction is famous or personally familiar faces minus unfamiliar faces. Although the strict inclusion and exclusion criteria we used in our ALE analyses resulted in a number of studies included in the final analysis that might be considered small [see Turkeltaub and Coslett (2010) for a discussion of group size], we used a conservative random effects ALE meta-analysis that allowed generalization of the results to the entire population of analyzed studies (Eickhoff et al., 2009). The results showed significant left-lateralized ATL activations for personally familiar and famous faces. Personally familiar faces activated distinct regions of the left superior ATL and the left orbitofrontal cortex. Regions activated by personally familiar faces showed some overlap with regions of the left temporal pole that were activated by famous faces. Famous face activations extended more ventrally, although not to the ventral surface. Other regions of overlapping activations are reported in Table 2.

Although we found robust activations to famous and personally familiar faces in the ALE analysis, the activations did not extend into the ventral ATL in and around the anterior MTG, as is commonly reported in monkey fMRI studies. There are several inter-related cognitive explanations for this difference: familiar faces may activate more superior/polar regions of the ATL compared to non-familiar faces (which are most commonly used in monkey studies), due to their emotional, semantic, or verbal content. Alternatively, the subtraction used in the studies that make up the ALE analysis is famous/familiar faces minus unfamiliar faces which essentially leaves behind non-visual information such as proper names, biographical information including unique information for the famous individuals, episodic information (e.g., experiences with that person), and affective information, especially for the personally familiar faces. Thus, the observed ATL activations represent some combination of these non-visual factors. For example, the fact that the ATL activations to familiar vs. unfamiliar faces were left-lateralized might imply that verbal factors such as the retrieval of proper names made an important contribution (Fukatsu et al., 1999; Tsukiura et al., 2002; Glosser et al., 2003).

A second more technical explanation is that neurons that are sensitive to familiar faces exist in the ventral ATL, but were not revealed by our meta-analysis due to signal drop out and distortion. Signal drop-out and distortion in the inferior surface and polar tip of the ATL are common and especially problematic when studies use a high TE and large voxel size. We selected studies for our ALE meta-analysis using strict inclusion and exclusion criteria in order to optimize our ability to reveal activations in the ventral ATL if present. However, we can not rule out the possibility that a majority of the studies included in our meta-analyses were still subject to these technical issues.

In order to address this interpretive problem and provide converging evidence for our results, we conducted an fMRI study of person knowledge using an imaging protocol that was optimized for coverage in the ATL. We examined activations to novel faces, famous faces, and personally familiar faces. Collapsing across all face conditions, faces contrasted to landmarks activated a network that included bilateral ATLs. When the conditions were split apart, it was found that a small region of the right ventromedial ATL was sensitive to novel faces, while bilateral ventromedial ATL activations were observed to famous faces as compared to the baseline task. To examine the specific contribution of familiarity, we contrasted familiar faces with unfamiliar faces. Personally familiar faces were associated with left lateralized activations in the orbitofrontal cortex, the insula, and the temporal pole. The polar activation began in the anterior STS and extended into superior aspects of BA 38. Famous faces activated a more restricted set of voxels in the temporal pole that overlapped with the activations to personally familiar faces but extended more ventrally.

Overall, the findings of the empirical study and meta-analysis were remarkably similar. Both studies showed a left-lateralized ATL sensitivity to two kinds of familiar faces. In addition, the only consistent overlap in activations in both studies to faces associated with knowledge was in the polar tip of the ATL. Optimized imaging parameters in our empirical study allowed us to see face-sensitive ATL activations in the ventral ATL, similar to what has been reported in studies of non-human primates.

### Functional subdivisions and anatomical localization

Several authors have suggested that the ATL has discrete functional subregions (Moran et al., 1987; Ding et al., 2009; Martin, 2009) and one such subregion may be a face selective region in the ventral ATL. fMRI studies in non-human primates have identified somewhat variable activation loci for faces, varying from the inferior bank of the STS on the lateral surface to the inferior surface of the ATL (Ku et al., 2011). In humans, two MVPA studies of facial identity using novel faces have also reported somewhat different loci in the right ventral ATL: one study reported an extremely medial peak in the uncus, possibly corresponding to perirhinal cortex (Nestor et al., 2011) while another study reported a peak in the anterior MTG (BA 21) (Kriegeskorte et al., 2007). Our activations to novel faces are remarkably similar to that reported by Nestor and colleagues. The most ventral ATL peak to unfamiliar faces in our study was at 29, −5, −31. The most ventral peaks to familiar minus novel faces in our empirical study were left-lateralized and in a similar depth plane to that reported by Kriegeskorte, but slightly more anterior in BA 38 (famous vs. novel faces: −32, 14, −36; best friends vs. novel faces: −47, 11, −31). The most ventral peaks found in the empirical study were to famous faces minus novel landmarks on the surface of the ATL at left (−36, 6, −42) and right (35, 3, −42) locations.

We did not have any a priori hypotheses about lateralization. It is plausible that the left-lateralized activations to familiar faces in both the meta-analysis and empirical study reflect lexical features of these stimuli that are not present in unfamiliar faces. We note that this is consistent with the neuropsychology literature reviewed earlier (Gainotti, 2007).

### The specific role of the anterior temporal lobe in person memory

There is widespread agreement that portions of the ATL play a mnemonic role in face processing, especially in face identification. It has been suggested that the ventral ATL face patch uses a population code to represent subtle differences between individual faces (Kriegeskorte et al., 2007). The exact facial dimension that the ATL uses to do this is not known. What we do know is that cells in the macaque ventral ATL respond to changes in facial identity but not to perceptual changes that leave identity intact, such as rotation (Eifuku et al., 2010, 2011). Similarly, the BOLD signal in ventral ATL, including perirhinal cortex, is sensitive to changes to facial identity but not other types of perceptual changes such as color (Graham et al., 2010).

These same ventral ATL regions are up-regulated in the presence of conceptual information about faces such as personal familiarity, semantic uniqueness, or names (Barense et al., 2011; Eifuku et al., 2011; Ross and Olson, 2011). Conceptual knowledge provides a powerful tool for abstracting over perceptual differences between items from the same class and highlighting differences between items from different classes. Conceptual knowledge, especially in the form of verbal labels, is a useful information-compression mechanism that allows us to carve up the perceptual world into the categories that are relevant to our behavior (Goldstone and Styvers, 2001). Interestingly, researchers in the aphasia and semantic memory literature have proposed that portions of the ATL have an important role in storing and retrieving concrete concepts although there is disagreement about the precise nature of this role and the ATL subregion involved in this process (Patterson et al., 2007; Simmons and Martin, 2009; Binder and Desai, 2011). Our laboratory and others have reported that the superior bank of the anterior STS/superior-polar tip is preferentially sensitive to social concepts such as social words (e.g., “friendly”) and vignettes that evoke theory of mind (Zahn et al., 2007; Ross and Olson, 2010). The superior loci for these effects may reflect the connectivity of superior-polar regions of the ATL to neuromodulatory regions, the amygdala, and hypothalamus [see Olson et al. (2012) for a review]. Indeed one recent study proposed that the dorsal ATL is part of an “affective” system while the ventral ATL is part of a social perception system (Bickart et al., 2012). Bringing these literatures together, it seems plausible that the ventral ATL codes for facial identity by linking-specific faces to social semantic knowledge stored in more superior ATL regions.

Cells in the ventral ATL can associate specific faces to identifying information such as affective feelings, names, and biographical information by virtue of their association formation capabilities. In the early 90's it was reported that single neurons in the ventral ATL of monkeys that initially responded to only one abstract pattern, would later respond to a second abstract pattern that had been associated via training with the first (Sakai and Miyashita, 1991). More recently it was shown that cells in the ventral ATL in monkeys can represent an trained associative pairing between faces and abstract patterns (Eifuku et al., 2010). Likewise, in humans it has been reported that successful encoding of person-related semantics with a proper name was associated with left ATL BOLD activity (Tsukiura et al., 2010). Even more compelling are findings showing that patients with left ATL lesions are unable to form new associations between names and pictures of objects (Sharon et al., 2011).

Although our discussion thus far has emphasized face memory, it should be noted that face patches in the ATL are sensitive to novel faces (Tsao et al., 2008). Our findings also show this. Based on the sensitivity of this region to novel faces, it has also been argued that the ATL is part of a network for perceptual face discrimination (Nestor et al., 2011). We agree with this sentiment however we argue that this is the proverbial “tip of the iceberg” since the properties of cells in this region appear to bridge perception and memory. Indeed, both components are required for accurate and rapid identification and there is a wealth of behavioral data showing that person identification is speeded by knowledge (e.g., Young et al., 1985, 1986; Bruce and Valentine, 1986). As noted earlier, cells in ventral ATL are only sensitive to certain perceptual manipulations such as changes in facial identity, but not to perceptual changes that leave identity intact (Eifuku et al., 2010, 2011). However, ventral ATL cells are acutely sensitive to different types of familiarity manipulations: responsiveness is enhanced by knowledge-base familiarity in the form of semantic knowledge (Nieuwenhuis et al., 2011; Ross and Olson, 2011) but decreased by perceptual familiarity in the form of stimulus repetition (Sugiura et al., 2001, 2011). The strong repetition suppression effect may underlie the familiarity signal reportedly lost after ATL damage (Bowles et al., 2007; Gainotti, 2007).

Although the response properties of cells in the ventral ATL might seem discordant, they in fact mirror important features of everyday experience with other individuals. On the one hand, when an individual is important to us we acquire knowledge about their name, interests, personality characteristics, and our emotional reactivity to them becomes more nuanced. On the other hand, there are individuals who we frequently see but we stop noticing, such as commuters on the 9 a.m. train, because they have no personal significance. Such *perceptual familiarity* is somewhat trivial when compared to the power exerted by knowledge on face identification. We propose that ATL face patches are active at both encoding and retrieval to integrate perceptual and mnemonic information to form a salience-tagged representation of different individuals.

### Conflict of interest statement

The authors declare that the research was conducted in the absence of any commercial or financial relationships that could be construed as a potential conflict of interest.
